# Science, Medicine and the Creation of a ‘Healthy’ Soviet Cinema

**DOI:** 10.1177/0022009418820111

**Published:** 2019-03-27

**Authors:** Anna Toropova

**Affiliations:** University of Nottingham, UK

**Keywords:** Children, Early Soviet Cinema, health, medicine, Socialist Realism

## Abstract

Cinema had long been hailed by Bolshevik party leaders as a crucial ally of the Soviet mass enlightenment project. By the mid-1920s, however, Soviet psychologists, educators and practitioners of ‘child science’ (pedology) were pointing to the grave effects that the consumption of commercial cinema was exerting on the physical, mental and moral health of Soviet young people. Diagnosing an epidemic of ‘film mania’, specialists battled to curtail the NEP-era practices of film production and demonstration that had rendered cinema ‘toxic’ to children. Campaigns to ‘healthify’ Soviet cinema, first manifesting in the organization of child-friendly screenings and forms of ‘cultural enlightenment work’, soon extended to attempts to develop a new children's film repertoire based on the results of psycho-physiological viewer studies. A vast variety of pedological research institutions established during the late 1920s and early 1930s began to experimentally test cinema's effects on children with the view of assisting the production of films that could cultivate a sound mind and body. Tracing a link between the findings of pedological viewer studies and the ‘healthy’ cinema championed in the 1930s, this article sheds light on the vital role played by medical and scientific expertise in shaping Stalinist culture.

In 1936, several years after Soviet filmmakers had been obliged to abandon experimental aesthetics in favour of a socialist realist ‘cinema for the millions’, the physician and film scholar Lazar’ Sukharebskii commended Soviet films for their positive influence on mental health. In contrast to the ‘traumatising’ impact that bourgeois cinema's sensationalist depiction of sex, violence and crime exerted on the minds of viewers, Sukharebskii argued, Soviet cinema's life-affirming narratives functioned as a powerful means of ‘psychoprophylaxis’, ‘vaccination’ and ‘preventive training’.^1^ Sukharebskii's sentiments were echoed by a range of Stalin-era cultural authorities who similarly professed that socialist realist cinema's optimism and heroic subject matter, as well as its conformity to classical principles (clarity, unity, decorum), rendered it a ‘healthy’ and ‘composed’ art form that was free from ‘hysterics’, ‘nervousness’, ‘convulsions’ and ‘psychological chaos’.^2^

The discursive conflation of socialist realist cinema and mental and physical wellbeing cannot solely be attributed to Stalin-era efforts to enforce compliance to a single aesthetic method. The attainment of a cinema that cultivated a sound mind and body had been set on the agenda by Soviet educators, medical professionals and scientific researchers well ahead of Joseph Stalin's ‘revolution from above’ (1929–32). Sukharebskii, the doctor who championed the ‘prophylactic cinema’ that filled Soviet screens by the mid-1930s, had, like many other medical specialists, spent much of the previous decade highlighting the myriad dangers that the films being screened in Soviet theatres posed to viewers’ health. Increasingly concerned about the damaging psycho-physiological effects of commercial cinema, early Soviet experts worked alongside film industry professionals to uncover ways of ‘making healthy’ both the practices of cinema-going and the films accessible to Soviet audiences. These collaborative investigations not only provide a lens through which socialist realism's origins can be better understood, but the complex lines of interaction between twentieth-century medicine, politics and culture. Expanding the parameters of the medicalisation process traced in recent histories of early Soviet Russia, an account of the post-revolutionary battle for cinema reform brings to light the role of medical and scientific expertise in establishing the ‘healthy’ aesthetics of the Stalin era.^3^

The aim of establishing a film industry that functioned to promote the physical, mental and moral wellbeing of the population, an objective already targeted by reformers in late imperial Russia, was certainly not exclusive to the Soviet Union.^4^ The harm inflicted by cinema on the health of viewers, particularly children and young people, became a key source of concern for educators, medical professionals, members of religious organisations and public authorities across Europe and the USA during the early twentieth century.^5^ Seeking to change the industry practices that had made cinema toxic to young viewers, reformers in Germany, France, Italy, and the USA initiated campaigns to transform the medium into a tool of edification. The transnational reach of efforts to healthify cinema and unlock its edifying power was attested by the opening of the International Institute of Educational Cinematography in Rome in 1928.^6^ Sponsored by the League of Nations and publishing its finding in five different languages, the institute sought to combat the influence of films that contradicted ‘the moral principles and traditions of civilisation’ and to promote the use of cinema for educational purposes.^7^

While having roots in pre-revolutionary campaigns, and sharing common features with initiatives abroad, the early Soviet cinema reform movement was underwritten by a revolutionary political agenda that endowed it with unique characteristics. Accounts of the psycho-physiological and moral harmfulness of the foreign-dominated film repertoire of early Soviet cinemas were inextricably intertwined with political anxieties about the ideological price of the Soviet state's sanctioning of small-scale private enterprise during the period of the New Economic Policy (1921–8). The drive to ‘healthify’ (*ozdorovit’*) Soviet cinema, coming into its own as the ‘tide of Westernism’ under NEP gave way to belligerent attacks on bourgeois culture during the cultural revolution, became invested with a political significance that pushed the parameters of cinema reform to encompass a radical restructuring of film industry practices not seen in other countries.^8^

The cinema's synonymity with modernity, communal experience and technological advancement made the medium a perfect ally of the Soviet mass enlightenment project. Party leaders like Leon Trotsky and Nikolai Bukharin waxed lyrical about the medium's capacity to replace vodka and religion in the lives of the masses and forge more enlightened and healthy forms of everyday life.^9^ By the mid-1920s, however, many Soviet educators, medical professionals and cultural authorities were concluding that cinema was exercising a ‘poisonous’ influence on its viewers. The vulnerable minds and bodies of children and teenagers were seen to be particularly at risk from contamination.

As Kara Ritzheimer has noted, heightened societal awareness of the potentially damaging effects of cinema on children was partly fostered by the early twentieth-century shift to an understanding of childhood and adolescence as distinct, pivotal developmental stages ‘during which young people were vulnerable to misguidance and corruption’ and required state protection.^10^ Paralleling trends in Europe and the USA, the study of child development – epitomized in the new scientific field of ‘pedology’ (or ‘child science’) – began to blossom in Russia at the beginning of the 1900s. Spanning disciplines including medicine, education, psychology and anthropology, this hybrid field took on even greater prominence after the October Revolution. By studying the psycho-physiological specificities of different stages of childhood, as well as the influence of different environmental factors on a child's development, pedology promised to determine the most effective strategies for cultivating a new generation of Soviet citizens.^11^ As part of their efforts to better understand the role of everyday life in the developmental process, pedologists and other medical professionals began to take an avid interest in children's leisure-time activities. Numerous studies conducted throughout the decade on the organization of children's free time left no doubt about the prominent place occupied by cinema in the lives of the younger generation.^12^

The results of a 1928 study conducted by the Department of Pedology at the Institute of Pedagogical Methodology (IMShR) indicated that cinema-going was far from an occasional treat for many Soviet young people. According to the study, 13 per cent of 8–17-year-olds in Moscow as well as the provinces frequented the cinema at least eight times a month, with a small percentage visiting more than 20 times. Pedologists reported with alarm that there were children in Moscow who practically spent their entire waking life at the cinema, arriving early in the morning and managing to sit through three or four screenings in a single visit, day in and day out.^13^

Noting that the denial of a trip to the cinema was accompanied by withdrawal-like symptoms of agitation and nervousness, Soviet educators and medical specialists concluded that cinema-going had become a destructive addiction. ‘Just like the alcoholic who cannot live without vodka or the drug addict who is dependent on morphine’, one pedologist mused, ‘film-maniacs are no longer able to live without the cinema’.^14^ Moreover, Soviet experts warned that they were not battling an illness that had claimed individual victims, but a country-wide epidemic that was ‘spreading by the hour and contaminating more and more of our Soviet young people’.^15^ Although ‘film-mania’ (*kinomania*) or ‘film-psychosis’ (*kinopsikhoz*) were not exclusively attributed to children and adolescents, the new generation of Soviet citizens was typically portrayed as the demographic group that was most at risk from this affliction.^16^

A heightened awareness of the dangers posed by film watching emerged in the context of a broader preoccupation with the health of Soviet youth. Several studies conducted in the mid-1920s by the Commissariat of Public Health (*Narkomzdrav*) – part of the wave of enquiries into the state of the population's health undertaken during this decade – revealed that thousands of Soviet school pupils in urban centres suffered from ailments such as anaemia, headaches, insomnia and spine deformity.^17^ Attributing the proliferation of such health complaints to the exhaustion of Soviet young people, doctors prescribed ‘active’ leisure activities in the open air and a healthy daily regimen that ensured children received 10-11 hours of sleep every night.^18^ Long cinema showings (increasingly the norm in urban centres), during which ticket holders sat in overcrowded, unventilated spaces late into the evening, could not be further from the ‘healthy leisure’ advocated by Soviet doctors.

In addition to bemoaning the ‘unhygienic’ conditions within crowded cinemas, specialists like Sukharebskii linked excessive film watching to various eye problems, ranging from mild eye irritation to swollen eyelids and conjunctivitis. A new film related eye disease was even diagnosed – ‘*kinematoftalmiia*’, or the deterioration of the iris attributed to frequent cinema-going.^19^ The warnings issued by Soviet specialists about the threat that the flickering of the screen, startling shifts from darkness to light and accelerated projection posed to children's eyesight mirrored Western European experts’ concerns about the links between film consumption and visual deterioration.^20^ Soviet doctors, however, were more careful to link negative physical symptoms to commercial practices of film demonstration that saw matters of health and safety subordinated to the pursuit of profit.^21^ With film going culture reviving under the New Economic Policy – conceived by Vladimir Lenin as a ‘tactical’ compromise with capitalism that would aid the country's recovery from economic collapse – theatre management was re-established as a money-making enterprise. Most cinemas during this period were either owned by film studios or ‘NEP-men’ – the new class of private entrepreneurs who profited from the relaxations on trade.^22^

The chief pedologist working on cinema at the IMShR, Abram Gel'mont, bemoaned that commercial theatres in Moscow had grown accustomed to screening multi-serial films in single showings that lasted as long as four hours.^23^ Such long periods within the auditorium, Soviet doctors warned, caused acute physical exhaustion:

‘the repeated process of physiological stimuli being sent to the visual cortex (located in the back sections of the cerebral cortex) via the optic nerve leads to brain fatigue, which commonly manifests in headaches, eye strain and other types of unspecified heaviness.’^24^

The IMShR warned that 42 per cent of 8–9-year-olds complained of headaches after screenings, while back pain and aching limbs affected 24 per cent.^25^ In the context of the epidemic of ‘exhaustion’ already diagnosed among Soviet youth, the revelation that cinema-going comprised 40 per cent of children's entire leisure-time activities was alarming indeed.^26^

Warnings about the damage that cinema-going exerted on the body were coupled with concerns about its stain on the fragile, developing psyche of children and adolescents. Based on the results of memory and attention tests, the IMShR concluded that a 90-minute screening placed more demands on children's mental alertness than an entire school day.^27^ The link between cinema and mental fatigue was particularly troubling given the perceived increase in nervous exhaustion (or ‘neurasthenia’) in early Soviet Russia, attributed by Soviet doctors to the great nervous stresses borne by the revolution, civil war and the demands of building the world's first socialist society.^28^ Medical and psychiatric examinations that were conducted at the Moscow psychiatric hospital and the clinic of nervous illnesses headed by the neurologist Grigorii Rossolimo found that nearly two thirds of frequent cinema goers were nervously ill, at the very least being afflicted with heightened levels of excitability and nervous sensitivity.^29^

At times Soviet pedologists echoed the concerns of cinema reformers abroad by pointing to the properties that rendered the cinema as such ill suitable for children. Cinema's presentation of a ‘condensed portrait of human life’ and its ‘fast tempo’ were some of the medium specificities linked by pedologists to the overstraining of the viewer's ‘memory, attention, and creative imagination’.^30^ It was much more common, however, for Soviet specialists to attribute ‘overstimulation’ specifically to Western productions and more commercially oriented domestic films.^31^

The commercial pressure that loomed large over *Sovkino* during the NEP period fostered a heavy dependence on foreign imports. Between 1923 and 1925, the years marking the zenith of the phenomenon contemporary commentators decried as ‘foreignitis’, over two thirds of the new films screened in Soviet cinemas were of Western origin.^32^ Another sizeable part of the repertoire was made up of domestic films that strove to compete with Western productions by mimicking their devices, such as the melodrama *The Bear's Wedding* (*Medvezh'ia svad'ba*, Eggert and Gardin, 1925). Routinely linking this type of sensationalist production to ‘psychological trauma’, educators and pedologists warned that the sleep disturbance and deprivation caused by over-stimulation rendered children apathetic, unparticipative and lethargic at school and reluctant to help out at home.^33^

The grave threat posed by overstimulation to a fragile, developing constitution was vividly brought to life in a 1930 book on cinema and youth written by Vladimir Pravdoliubov, another prominent pedologist at the IMShR. Impressing the ease with which a child with no previous history of mental or physical illness could be transformed into a ‘film-invalid’, Pravdoliubov cited the case of a 14-year-old Moscow schoolboy whose behaviour at home and at school suddenly became erratic and uncontrollable.^34^ Teachers at the teenager's school, Pravdoliubov recounted, were shocked to find him arriving to class in a highly agitated state, inciting violence towards his schoolmates and making a habit of sitting under his desk. Several months of such behaviour culminated in the boy stabbing a fellow pupil. An investigation exposed the teenager as a ‘film maniac’ who had severed ties with his family and spent his evenings at the cinema. The youth expressed boredom with his surroundings, hatred of school, a desire to flee to the USA and intentions to murder his mother. A psychiatric examination diagnosed an acute case of neurasthenia and general nervous distress, prescribing urgent admission to a treatment facility. Pravdoliubov noted that the patient's symptoms improved as soon as he stopped frequenting the cinema.^35^

Pravdoliubov's case study makes explicit how ‘film mania’ personified the threat that NEP-era practices of film distribution and demonstration posed not only to the mental and physical wellbeing of Soviet young people, but to their moral and political consciousness. As a number of scholars have shown, the conflation of physical and mental degradation with political deviation was a common feature of anxieties about the state of the younger generation's health that burgeoned during the period of ‘state capitalism’. Symptomatic of what Kenneth Pinnow has recently framed as a medicopolitics targeted at the elimination of ideological pathology, early Soviet medical diatribes against the dangers of western cinema framed disease as both physiological and ideological.^36^

Cinema's great technological achievements, Gel’mont warned in 1929, were being transported to the Soviet Union alongside ‘all the filth and degeneration of the foreign capitalist world’. ‘All the evils of capitalist everyday life’, he continued ‘are reflected in the film products being sent to us from beyond Soviet borders’.^37^ The results of the 1928 IMShR survey of children's film-going habits revealed that a significant percentage of Soviet young people much preferred films depicting the lives of wealthy foreigners to those narrating the struggles of Soviet workers.^38^ Pravdoliubov noted that exposure to such films made the younger generation liable to perceive their everyday existence as ‘grey’ and ‘impoverished’ in contrast to the ‘wealth’ and ‘luxury’ of the Western world.^39^

Concerns about the prospect of a country-wide ‘film mania’ epidemic extended far beyond the domain of pedological expertise. Regional and national newspapers (including *The Workers’ Newspaper* and *Evening Moscow*) began to cover this worrying trend.^40^ The results of studies carried out by the IMShR even made their way onto the pages of *Pravda*. Citing Gel’mont's worrying statistics, the official organ of the Communist Party conjured the frightening image of ‘hundreds of thousands’ of ‘nervous’ and ‘disorderly’ ‘film maniacs’ residing in urban centres across the Soviet Union.^41^ The many types of morally and politically suspect behaviour blamed by the popular press on ‘film mania’ ranged from relatively minor deviations from Soviet ideals – educators bemoaned school children who badgered their parents for cinema ticket money and teenagers whose performance on the factory floor and dedication in school had been compromised by daydreams about Hollywood stars – to more perturbing forms of political disengagement such as attempts to flee abroad in pursuit of the ‘better life’ depicted onscreen and involvement in criminal activity.^42^ Appearing at a time of heightened anxiety about the ideological corruption of Soviet youth effected by the partial revival of market economy conditions, the film maniac became a symbol of the dangerous effects of increased exposure to a non-communist world-view.

The link between excessive film consumption and criminality became a particular favourite of the NEP-era press. Alongside lamenting children's resort to petty theft to obtain money to fund a ‘film addiction’, newspapers began to feature sensationalist accounts of Harry Piel fans who threatened girls with rape if they did not hand over the price of a cinema ticket and teenage film maniacs who did not stall at committing murder in pursuit of their poison.^43^ One frequently cited case, first reported in *Evening Moscow*, featured a 15-year-old youth who funded a late-night binge of back-to-back screenings at multiple cinemas across the city by strangling a young boy and stealing his coat. The method of strangulation used by the ‘film-addict’, the paper reported, mimicked one that had featured in a recent film.^44^

To be sure, press reports of children imitating violent onscreen acts – readers of one particularly gory account were informed that a trip to watch the Soviet 1924 educational film, *Abortion* (*Abort*, Galkin and Lemberg), resulted in a gang of boys attempting to perform the operation on a cat – tapped into concerns about cinema's ‘suggestive power’ that were prevalent across early twentieth-century Europe.^45^ The frequency with which press reports lamented young people's proclivity to deviate from Soviet standards of behaviour due to their ‘overidentification’ with Hollywood protagonists, however, points to a specifically Soviet anxiety about the contaminating threat of a ‘foreign’ ideological system.

‘American film tricks cultivate Russian hooligans’, pronounced a local newspaper in December 1927. Blaming spectacular American adventure films like the Douglas Fairbanks vehicle, *The Thief of Bagdad* (Walsh, 1924) on an epidemic of hooliganism and anti-social behaviour among schoolboys, the journalist lamented:

After these sorts of American films our young children ‘exercise’ boisterously out on the street, in the garden, at home, and in school. They practice on their brothers, sisters, comrades and passersby. Just the other day, for example, a crowd of school-age children on Sovetskaia street could be seen throwing themselves at fellow children passing by. As a result of this ‘film-attack’, a pupil split her head open and an onlooker broke her back.^46^

Exposure to Western films was similarly attributed to an awakening curiosity in matters that should have been irrelevant to Soviet young women – ‘gowns, powder, manicures, and painted lips’.^47^ As Anne Gorsuch notes, Bolshevik moralists decried the alarming numbers of young female workers who were squandering their salaries on the imitation of Western screen idols.^48^ The ascetic standards of sexual morality advocated by many educators and Komsomol activists were also felt to be at risk from Western productions. The eroticism of foreign films, Soviet experts warned, was liable to arouse an untimely interest in sexual matters and turn the darkness of the cinema hall into a sanctuary for deviant sexual practices.^49^ Even communist children's organizations felt powerless in the face of this threat. Concluding that cinema led children to take an interest in sex earlier than normal, one pedagogue noted that there was not a single screening attended by her brigade of Young Pioneers where a case of sexual excitement among older boys was not witnessed.^50^ Cinema's impact on the sexual morality of women was a still greater cause of anxiety. P.I. Liublinskii, a Soviet legal scholar who explored the link between cinema and criminality in a 1925 book, professed that foreign films had a role to play in driving Soviet young women to prostitution. A trip to the cinema was not only liable to awaken a desire for the ‘glamorous and easy life’ depicted in foreign films, he warned, but also to stir ‘nervous and sexual excitement’ than could overwhelm ‘female reserve’, leaving Soviet young women vulnerable to solicitations on exit from the theatre.^51^

Prostitution, which underwent a steep rise in early Soviet Russia as women workers disproportionately bore the brunt of unemployment, was not the only NEP-era social problem that the cinema came to be associated with. Pedagogues also noted that Soviet cinemas had become a hub for homeless street children (*besprizorniki*) – Civil War orphans who became a potent symbol of the state's struggle to meet social demands during NEP.^52^ Commercial film theatres were described as a magnet for gangs of delinquent youths who crowded the surrounding streets, bummed cigarettes, fought among each other, attacked passers-by, and led ‘pornographic conversations’.^53^ Attendance at commercial cinemas and engagement in practices that, while due to be eradicated under socialism, still persisted in NEP-era Russia came to be routinely conflated. Alongside the tavern and the dancehall, the cinema became synonymous with a seductive NEP urban landscape where non-communist ways of life were able to continue unabated. A nightmarish place of ‘shoving, screams and swearing’, the cinema foyer was described as a breeding ground of moral and physical decay.^54^ The young people who crossed this threshold were bombarded with the sight of sensational posters featuring half naked damsels in the clutches of bestial kidnappers, the sound of ‘bourgeois’ foxtrots and gypsy ballads, and the heady smell of cigarette smoke.^55^

For all their vocal warnings about the damage to children's physical, mental and moral wellbeing effectuated by unbridled exposure to unsuitable films, Soviet educators and pedologists never questioned that the cinematic medium held unrivalled capacities to healthify the body politic. Even state officials like the Soviet Commissar for Health, Nikolai Semashko, were vociferous in pronouncing cinema ‘as one of the most powerful means of making the population healthy’.^56^ Unlike in Europe and the USA where the cinema reform movement was characterized by both ‘negative’ and ‘positive’ approaches (the former stressing censorship and access restrictions and the latter targeting cinema's use for the purposes of edification), Soviet educators and pedologists almost unanimously took a pro-cinema line.^57^ Soviet specialists were careful not to indict cinema as such, but a commercially oriented film industry whose pursuit of profit had resulted in lax censorship, overcrowding and excessively long showings. If the existing system of film production and film distribution could be transformed, pedologists argued, cinema's damaging effects could be ‘minimised and even completely eradicated’.^58^

While specialists in other countries also began to recognize cinema's ‘transformative possibilities’, Soviet doctors’ unwavering faith in cinema as a means of prophylaxis reflected Bolshevism's distinctive emphasis on culture's pivotal role in the revolutionary process.^59^ Soviet ideologues and cultural producers were united in seeing cultural technologies like the cinema as vital tools of instilling a new revolutionary consciousness and creating a New Soviet Person. The battle for a cinema that would create a new healthy population paralleled a variety of other cultural initiatives that targeted the creation of a new ‘higher’ human type, including the ‘life-building’ programmes of the Soviet avant-garde and cultural enlightenment campaigns.^60^

A variety of measures to ensure cinema's ‘transformation from a factor that demoralized the child's psyche into a valuable pedagogical tool’ were discussed and enacted in the 1920s.^61^ One ‘antidote’ advocated by educators and pedologists in Soviet Russia, as by cinema reformers elsewhere, was the restriction of children's access to cinema and the ‘healthification’ of the existing film repertoire through censorship.

In 1923, the administrations of larger cities within Soviet Russia enacted ‘obligatory orders’ which forbid children under 8 years of age access to film theatres and limited the admission of under-16s to screenings that were specifically intended for children.^62^ By the mid-1920s, however, it had become apparent that such local decrees were not being strictly enforced. Complaints were raised that inspections and checks were periodic even in central areas and did little to curb access to inappropriate films in second-tier cinemas on the outskirts of the city, where children continued to make up as much as half of the clientele.^63^ While the proprietors of local cinemas were liable to receive a fine for admitting under-age children, this made only a small dent in their finances. Moreover, the enforcement of fines was subject to the discretion of individual police officers, who rarely took the issue seriously.^64^ Censorship efforts tightened considerably towards the end of the 1920s. A special commission for the ‘pedagogical censorship of films’ was established by the Commissariat of Enlightenment in 1927. The commission required every newly released film to be issued with a ‘passport’ that specified its appropriateness for different age ranges. Those film theatres shown to be disregarding these instructions were to be fined.^65^ In 1928, censors began to crack down on films that depicted ‘prostitution’, ‘debauchery’ and ‘criminal activity’ in an uncritical manner.^66^

Even as legal experts, health professionals and educators called for more rigorous controls, referencing the restrictions on children's access to cinema enforced by other Western European countries, they recognized that overly strict laws and bans would not be conducive to the task of channelling the medium in the service of cultural enlightenment.^67^ Cinema's healthification, they argued, would only be achieved if restrictions on access were coupled with efforts to offer children attractive alternatives.^68^ One important means through which pedologists and educators sought to transform cinema-going from an anti-social activity to an educational practice that inculcated healthy habits and customs was the organization of matinée screenings for children. Held at local cinemas in the morning or early afternoon under pedagogical supervision, children's screenings facilitated the selection of age-appropriate films and the censorship of scenes deemed unsuitable by experts. To distinguish them from evening showings targeted towards adults at commercial theatres, organizers strove to make these matinées free or, at the very least, not to charge viewers more than 10 kopeks. They also offered discounted entrance fees to groups and school collectives as a means to remedy young viewers’ proclivity to visit the cinema as ‘disorganised’ and isolated consumers.^69^

Children's screenings presented an opportunity for educators to closely monitor children's behaviour within the theatre and to eradicate undesirable habits and behaviours. Under the vigilant gaze of the instructor, viewers entered the building in orderly pairs and took up their seats according to height. Any ‘force, shoving or fighting’ was strictly forbidden. Turning the cinema into an instrument of social integration, educators’ control over seating arrangements checked girls’ and boys’ instincts to divide into same-sex groups and curbed the tendency of children of different nationalities to sit apart from each other. Silence was observed during the screening and the consumption of snacks banned.^70^ Young viewers entering the new children's cinemas that began to open in Soviet Russia from 1928 were similarly confronted with a stringent list of behavioural rules. Smoking, spitting, littering and fighting were prohibited, with perpetrators risking eviction and bans.^71^

Children's screenings were also envisaged as a means of ensuring the observance of ‘hygienic norms’ designed to ‘neutralise’ the harmful physiological effects of cinema visits. Alongside ensuring that the duration of screenings did not exceed recommended guidelines and that their frequency was limited to weekends and celebration days to minimize fatigue, organizers sought to comply with a comprehensive list of preventative health measures drawn up by Soviet doctors. The building used for film demonstration was to be aired regularly and the number of children admitted was not to compromise the 1.25 metres of space required for each viewer.^72^ Steady projection at a slightly slowed down tempo without flickering was to ensure that children did not resort to ‘excessive strain’ to understand the film. The screen was to be large, evenly illuminated and positioned at least 5 meters away from the front row. Organizers were also to ensure that the screening room was pitch-black and to avoid stark transitions from darkness to light.^73^

The organizers of children's screenings transformed the cinema foyer into a calming space where ‘occasions for excitability and nervousness’ were eradicated.^74^ Children's orchestra performances replaced the ‘bourgeois’ music that resonated through the foyer during evening showings. The sensationalist advertising that greeted spectators at evening screenings was exchanged for wall newspapers, children's artwork and new age-appropriate film posters designed to not only capture the attention of young viewers but to ‘guide’ the viewer's mode of reading and set out a clear path of interpretation.^75^ Demonstrating the attempt to turn a space previously associated with NEP-era indulgence into a venue of aesthetic education, refinement and cultured behaviour, children's cinema manuals instructed organizers to furnish theatres with coatrooms, decorative plants and buffets serving hot drinks.^76^

Different types of ‘cultural enlightenment work’ conducted before, during, and after children's matinées targeted the creation of a new, active mode of viewership.^77^ Circling the foyer prior to screenings, ‘cultural enlightenment workers’ were tasked with assisting viewers’ comprehension by flagging the upcoming film's central themes and illuminating its social, economic and political contexts. In the case that Western films were shown, the cultural enlightenment worker strove to educate a critical perspective on the narrative by drawing attention to the social problems and class conflicts evaded on screen.^78^ Reflecting the growing cult of physical culture (*fizkul’tura*) in early Soviet Russia, some children's film theatres endeavoured to turn child spectators from ‘passive observers into active participants’ by premising film screenings with 10–15 minutes of coordinated physical exercise, games, and rhythmic dance routines.^79^

Before commencing the film demonstration, the teacher or cultural enlightenment worker delivered an introduction that briefly familiarized viewers with the film's subject matter and characters, provided any necessary historical and geographical context and pre-emptively clarified any difficult moments. The supervisor was also responsible for reading out subtitles during the screening (a measure that was seen to have a ‘calming influence’ on the audience) and for explaining any content that proved difficult for children to grasp. Educators would close the film demonstration by initiating a public discussion, asking viewers to put the film's characters on ‘trial’ or chairing a debate on the issues raised by the narrative.^80^

Articles depicting the work of the first Children's Cinema (*Detkino*) in Moscow, which opened in 1928 in the Sukharevskii district of the city, were effusive in praising its ‘healthifying influence on the organisation of children's leisure’.^81^ A 1929 report on a *Detkino* screening of a revolutionary film described a radically transformed form of recreational activity; having being prepared for the screening by explanatory conversations and visuals, *Detkino*’s viewers followed the screen as ‘active participants’. They greeted the toppling of autocracy shown on screen with cheers and a rousing rendition of the ‘International’. After the screening, young viewers participated in art circles, registered their impressions from the film in colourful displays to be put up in the foyer, worked on contributions to the wall newspaper or read up on Lenin's biography and the history of the revolution in the library corner.^82^ Praising the state expansion of the *detkino* network in the early 1930s, Stalin-era officials claimed that these child-friendly spaces had proved victorious in the battle against hooliganism and delinquency.^83^

Attempts to transform the cinema into a ‘*kino-shkola*’ (cinema-school) that increased viewers’ ‘level of culturedness’ and inculcated ‘orderly habits’ through the organization of matinée screenings and the opening of children's film theatres was part of a broader drive to co-opt cinema for the purposes of education.^84^ A campaign for the ‘cinefication’ of Soviet schools, preceded by the ‘mass film lessons’ than had been held in Moscow cinemas from the mid-1920s, was in full swing by the end of the cultural revolution.^85^ The state not only began to supply schools with projectors to facilitate the screening of films in lessons but invested in the creation of educational films for children, including sponsoring research designed to help directors ensure the optimal effectiveness of their productions.^86^

The organizers of children's screenings and ‘cultural enlightenment work’ readily acknowledged that such attempts were largely interim measures to compensate for the lack of Soviet films that were suitable for young viewers. The most effective way to combat ‘film-sickness’, pedologists and educators argued, was to replace the ‘disorganising’ and ‘demoralising’ films shown in Soviet cinemas with healthier alternatives.^87^ To be sure, a limited number of domestic children's films were approved by Soviet educators and studied in detail by pedologists. Among the productions most frequently singled out for acclaim were *Golden Honey* (*Zolotoi med*, Petrov and Beresnev, 1928), a re-education drama set at a labour commune for juvenile delinquents; *Van’ka and ‘Avenger*’ (*Van’ka i ‘Mstitel’’*, Lundin, 1928), a film narrating the embroilment of a boy and his canine companion in Red Army assignments; and a 1927 adventure focusing on the exploits of a Civil War orphan, *Ania* (Preobrazhenskaia and Pravov). These singular achievements, however, did little to damper educators’ complaints about the underdeveloped and sporadic character of Soviet children's film production.^88^

The imperative of accelerating the creation of home-grown children's films was made more pressing by the cultural, social and economic upheavals of the late 1920s and early 1930s. The New Economic Policy's displacement by a frantic drive to ‘build socialism’ through breakneck industrialization, forced collectivization of agriculture and cultural revolution under Stalin (1928–32) pushed Soviet film production to become self-sufficient and to radically curb its dependence on foreign imports.^89^ Soviet health and education experts were not only called upon to cultivate new practices of film spectatorship but to help create a brand new film repertoire that could wholly replace ‘damaging’ films.

The production of films that could cultivate citizens healthy in mind and body was a project that the Soviet film industry was not thought to be capable of accomplishing alone. Pedologists and psychologists were to play no less of an important role in this process than the filmmaker. As Pravdoliubov asked in a 1927 article, how would Soviet directors possibly manage to create successful films for children without any knowledge of young viewers' psycho-physiology, the characteristics of their thinking, perception, attention, imagination, or their needs, interests and demands?^90^ A range of research cells and laboratories founded in the 1920s began to address these questions, accumulating medical and scientific knowledge about the child spectator. Relied upon to provide practical instructions and advice to Soviet film producers, health professionals took on an unprecedentedly prominent role in the transformation of film industry practices.

Investigations into the child spectator were part of a broader drive to better understand the mechanics of film spectatorship with recourse to the expertise of medicine and the psy-professions.^91^ A number of research cells called on the techniques of psychologists, reflexologists and neurologists to unpack the effects of films on the psychophysiology of adult viewers. The ‘laboratory for the study of mass behaviour and mass psychotechnics’ at the Polytechnic Museum in Moscow drew on the ‘latest research from the field of psychoneurology’ to investigate the effects of Soviet mass culture on its recipient. Headed by the psychiatrist Pavel Karpov, the laboratory enlisted Dr Konstantinovskii (a neurologist who had previously worked alongside Vladimir Bekhterev) to map the types of reactions (‘emotional’, ‘motor’, ‘unconscious’) produced by different forms of mass culture.^92^ In 1928, the laboratory appointed the leading film director of the Soviet avant-garde, Sergei Eisenstein, to head its research into the psycho-physiology of the viewer. Eisenstein's research programme called for the audience effects of a variety of film–stimuli to be tested in a laboratory setting by reflexologists.^93^ The film commission at the State Institute of Art History in Leningrad similarly sought to forge ties with centres of reflexological research, including the Institute for the Study of the Brain, in its quest to understand spectator reactions.^94^

Initiated at a similar time as the Payne Fund's investigations into the ‘nature and extent’ of cinema's influence on children in the USA, as well as the International Institute of Educational Cinematography's enquiries into cinema's ‘moral and social influence’ on children, research on the child viewer quickly became a prominent subfield of Soviet psycho-physiological film research.^95^ Opening in 1926, the pedological film laboratory at the IMShR quickly became one of the most prolific centres of child-focused viewer research in Soviet Russia. The laboratory was staffed by pedologists including Gel’mont and Pravdoliubov, equipped with a wide-array of psycho-physiological measuring devices and furnished with a film screening room.^96^ Setting itself the task of identifying the types of film form and content that were the most conducive to exerting a pedagogical influence over the spectator and facilitating a ‘normal’ process of child development, the laboratory pursued two main lines of investigation. Firstly, the laboratory's pedologists sought to identify the different tastes, imaginative capacities, and modes of spectatorship that pertained to children from different social backgrounds and age ranges. Through questionnaires, investigators probed into what attracted different types of children to particular films and what they liked and disliked about the existing repertoire of Soviet cinemas. Viewer observations as well as post-screening interviews and psychological tests were used to shed light on the distinct ways that younger and older children, girls and boys, the offspring of blue-collar and white-collar parents engaged with films.^97^

Secondly, at the behest of the Soviet film industry, the laboratory sought to experimentally test the psycho-physiological effects of cinema on child spectators.^98^ Special attention was devoted to the study of emotional impact. While pedologists identified heightened affective excitability as a major symptom of ‘film-sickness’, they were also cognizant that cinema's capacity to compel intense forms of emotional engagement formed an integral part of its educational power. The accumulation of knowledge about the types of emotions cinema elicited, the strength and depth of these reactions, and their impact on children's comprehension was thus to help Soviet filmmakers deploy emotion ‘in the correct way pedagogically’.^99^

Much like the Payne Fund's research into cinema's impact on children's emotions that centred on the measurement of heartbeat, blood pressure and galvanic skin response, Gel’mont's studies understood emotion as primarily a physiological phenomenon.^100^ Using biomedical equipment including the pneumograph and sphygmograph, the IMShR laboratory monitored the precise bodily changes impelled by four different types of stimuli: a clip from a detective film starring Harry Piel, a revolutionary film, a documentary and an ‘erotic film’. The laboratory also began to trial the use of a galvanometer to monitor levels of affective arousal by measuring alterations in skin conductance. Conflating the mental and the physiological, the laboratory translated quantitative alterations in breathing rate, pulse, and rhythmic movement into qualitative emotional changes.^101^ Modifications in the viewer's facial expressions, captured during moments of heightened dramatic tension on photographic film, were similarly taken as indicators of the child's psychological state.^102^ While these methods were indicative of the laboratory's physiological bias, the research cell was not completely insensitive to the psychological aspect of the film experience. Comprehension questions as well as a task asking viewers to sort photographic film stills into narrative order were used to investigate the impact of emotional arousal on the child's understanding. As a means to uncover the specific elements of the film that had most vividly captured the child's imagination, investigators also asked the viewer to write a composition about the watched material after the screening.^103^

In addition to the question of emotional excitability, the laboratory was interested in uncovering cinema's impact on children's mental alertness and work capabilities. Alongside determining the levels of physical exertion demanded by a film screening via a handgrip test with a dynamometer, a ‘graphic tremometer’ designed by the US behaviourist Edward Thorndike, and a spirometer to record vital capacity, the IMShR sought to evaluate the demands that different films placed on the viewer's mental faculties. The laboratory's investigators deployed a two-part test for mental exertion (devised by one of the fathers of Russian pedology, Aleksandr Nechaev) that checked for a lack of coordination between a child's sensory processing capacities and their fine motor skills. The first task tested children's levels of attention and memory retention by asking subjects to write down all the numbers they could remember from a spoken list of 12 double-digit figures. The corresponding test for motor skills gave subjects 30 seconds to write down as many possible numbers in numerical order from a random starting point provided by the investigator.^104^ Applying Nechaev's methodology to the study of a film's effect on viewers, Pravdoliubov diagnosed children whose writing speed was lower than their level of memory retention as suffering from the nervous disorder of psychasthenia, and those whose performance in the motor activity task was better than in the sensory skills assessment as victims of neurasthenia.^105^ Expanding Pravdoliubov's initial investigation, the laboratory proceeded to compare the levels of fatigue that resulted from film watching with those of school attendance. Testing four different types of films at weekly intervals, the laboratory's pedologists subjected a group of school pupils to the Benjamin Bourdon test for attention and mental alertness before and after a school day, and at three points during the ensuing cinema visit.^106^

The question of how to bring cinema into line with pedological requirements was addressed at other research centres, including the Institute of Psychology, Pedology and Psychotechnics (formerly the Moscow Institute of Experimental Psychology), the Krupskaia Academy of Communist Education and the Institute of Extra-Curricular Educational Activities Methodology (IMVR). The cinema commission at the IMVR became a site of particularly intensive research into the children's film properties that could best serve ‘the tasks of communist education’.^107^ Under the leadership of the pedologist Nikolai Zhinkin, the institute practiced testing different edits of the same film on select group of viewers as a means to determine the child-friendliness of different narrative forms and stylistic devices.^108^ Unlike the pedological film laboratory at the IMShR, the IMVR's cinema commission was primarily interested in the question of psychological effect, relying on qualitative methods such as audience observations, interviews and children's compositions. Developing close ties with Soviet film production, the cinema commission was routinely called to provide expert advice on scenarios, scripts, completed films and thematic plans for the film studios *Mezhrabpomfil'm* and *Sovkino* (and later, *Soiuzkino*). The collaboration between IMVR researchers and industry professionals also resulted in the production of a manual on how to write screenplays for children as well as in the creation of short films that were used to test the success of different narrative and stylistic strategies with young audiences.^109^

While most Soviet research on child viewers focused on school-age children, cinema's impact on pre-schoolers was also taken into account. Made up of pedologists, paediatricians and film technicians, a research collective headed by M. A. Polman at the Baumanskaia film station in Moscow sought a better understanding of how under sevens engaged with cinema. The research brigade ran test screenings at 40 local kindergartens in order to identify how filmmakers and exhibitors could render cinema safe for the youngest Soviet viewers.^110^ The effects of different films on children's comprehension, nervous systems and energy levels were gauged through close observations of individual viewers and small groups during screenings, as well as during post-screening discussions, games and drawing exercises. To evaluate the accessibility of various film excerpts, investigators posed a series of comprehension questions to their subjects, studied how they had interpreted the screened material in their drawings and asked children to recount what they had seen a day after the film demonstration. Eyesight tests and medical examinations performed in the middle of the screening and at its conclusion checked for signs of physical fatigue.^111^ In addition to monitoring select children in their home environment, the research brigade sought to uncover cinema's impact on children's sleep patterns through night-time observations.^112^

The Baumanskaia research project sought to challenge the widespread presumption that cinema was unsuitable for 7–8 year-olds. Instead of testing the impact of films intended for adults on pre-schoolers, the investigators used six 20-minute films that were specially adapted from full-length features. A stringent set of guidelines was followed to make sure that the film demonstration process accorded with standards of health and hygiene.^113^ Focusing their investigation on age-appropriate films and controlling the process of demonstration, the Baumanskaia researchers obtained results that directly contradicted the Payne Fund's findings that children were more restless than normal after a visit to the cinema. In the majority of cases, the researchers concluded, cinema had no ill effect on children's sleep patterns or behaviour at home.^114^ Showcasing their investment in the project of unravelling cinema's positive impact on mind and body, the Baumanskaia collective also drew attention to their preliminary findings that their screenings had a calming effect on children with behavioural problems.^115^

In contrast to many of their counterparts abroad who were reluctant to identify with any objectives beyond the advancement of scientific knowledge about cinema's effects, Soviet film pedologists saw themselves as active participants in the project of cultivating mentally, physiologically and ideologically sound citizens.^116^ Soviet researchers’ concern to spotlight the healthifying power of cinema was perhaps best exemplified in the studies of child film viewers conducted at the Institute of Psychology, Pedology and Psychotechnics in the early 1930s. Incorporating investigations of deaf mutes and sufferers of neurological disorders such as aphasia into its study on children's perception and understanding of different forms of film art and editing, the institute's researchers sought to harness cinema's potential to transform ‘defective’ children into ‘bona fide builders of socialism’.^117^

Many prolific centres of viewer investigation, including the IMVR and the IMShR, were closed down at the beginning of the 1930s. Studies of child spectatorship survived the cultural revolution, however, coming to a standstill only when the discipline of pedology was officially attacked in 1936.^118^ The pedologists who had studied viewer responses at the IMVR and IMShR in the 1920s (Gel’mont, Pravdoliubov and Zhinkin) resumed their investigations at other institutions in the early 1930s. Taking up a post at the ‘sector for the study of film perception’ at the Higher State Institute of Cinematography (VGIK), Zhinkin led an investigation into the ‘effectiveness’ of educational films for children under the sponsorship of the Soviet educational film trust *Soiuztekhfilm*. Using psychological observations and tests, the scholar studied the impact of thematic content, formal construction and visual means of presenting information on student comprehension.^119^ VGIK also became the site where the IMShR researcher, Pravdoliubov, continued his work on the psycho-physiological fatigue caused by film watching.^120^

Gel'mont, in turn, took up a post within the ‘Children's Cinema Workshop’ at the Central House of Children's Aesthetic Education (TsDKhVD) in the early 1930s. This research centre employed doctors, pedologists, psychologists and other specialists to help Soviet film organizations with all questions relating to the production of children's cinema. With the view of formulating the ‘pedological and pedagogical standards’ with which Soviet children's films were to comply, Gel'mont's department investigated cinema's impact on children's eyesight, nervous system and behaviour in its experimental research laboratory.^121^ Acting as a consulting and supervisory body, the Children's Cinema Workshop helped film organizations to test out their productions on audiences and to assess the pedagogical-pedological appropriateness of their outputs. The centre's staff took part in the compilation of film studio thematic plans, organized conferences of children's film writers and directors, reviewed completed screenplays and filmed material, and sent out ‘methodological bulletins’ to film organizations.^122^

Another major way in which the research centre aided Soviet film production was through educational training. Gel'mont was responsible for running an extended training course on ‘cinema and pedology’ for Soviet educators and screenwriters. Introducing students to the tenets of pedology, the course outlined the importance of taking the psycho-physiological characteristics, and mental and sexual development of target age groups into consideration when making children's films. It similarly acquainted film producers with pedological findings on the mental fatigue caused by cinema and its influence on the nervous systems of different age groups.^123^

The pedological spectator research that was conducted in the late 1920s and early 1930s began to set out the specific ways in which Soviet filmmakers could ensure that their films had a positive effect on the young generation's mental and physical development. These guidelines sought to steer the directors of Soviet children's films on a path that not only eschewed the sensationalism of bourgeois productions, but also rejected the experimentation and abstraction of avant-garde cinema. In an implicit critique of the type of challenging, experimental works that the pioneers of Soviet montage cinema produced in the 1920s, pedologists stressed the importance of making concessions to the viewer. Research findings that clearly connected cinema visits to mental and physical fatigue led many pedologists to spotlight the importance of easing the demands that filmmakers placed on the viewer's attention and concentration. Instructing children's film producers to observe the principles of clarity and accessibility, researchers stressed the need to guide the viewer's attention through narrative signposting, clear shot composition and the use of visual aids such as close-ups and subtitles.^124^ The use of ‘unusual’ film techniques such as accelerated motion and expressive framing was not recommended. ‘Unusual and striking camera angles, which may be effective in and of themselves, should be abandoned for the sake of clarity and accessibility’, contended Zhinkin.^125^ Symbolism was similarly rendered off limits; pedagogues noted that the type of abstract allegory that featured in films like Eisenstein's *October* (*Oktiabr’*, 1928) led to boredom and fatigue.^126^

Specialists unanimously warned that a rapid pace of editing and persistent cross cutting between different narrative lines was liable to overburden and disorient young viewers. ‘A rapid change of episodes forces children to constantly shift their attention, tiring and agitating them greatly’, concluded the Baumanskaia research brigade.^127^ In addition to advocating a measured pace of editing and longer takes, pedologists pointed to the error of films where fragmentation and disjointedness prevailed over synthesis and coherence. Only films with logically developed narrative lines, clear plot markers and easily identifiable protagonists, pedologists claimed, could maintain the viewer's attention and ensure sound understanding.^128^ Pedologists also warned about the perils of overlooking another key factor in audience comprehension – emotional engagement. A film that exerted a positive pedagogical influence, Zhinkin noted, was a film that acted on the emotions and did not ‘leave the viewer cold or indifferent’.^129^ Films for children were to be ‘cheerful, life-affirming’ and filled with ‘optimism’, shunning ‘everything that was antisocial, crude or foul’.^130^ Finally, pedologists stressed the importance of realism. Films that were ‘realistic’, rather than ‘removed from life’, Pravdoliubov claimed, would help to cultivate a ‘healthy and lively person’ rather than ‘an unhealthy type of child, a sick fantasist, a psychopathic drug addict, who is closed off from real life and lives in a word of images’.^131^

While it is difficult to gauge the precise extent to which investigations into children's tastes and capabilities initiated in the 1920s shaped filmmaking practices, the Soviet film industry's engagement with pedological research is indisputable. Conceptualized as an integral part of the process of raising the quality of Soviet children's film production, pedological viewer research strove to directly influence the work of film organizations.^132^ The establishment of sound lines of communication between researchers and film industry personnel became a top priority, particularly in the early 1930s when state support for any research venture that could not demonstrate immediate practical implications began to dwindle. Research cells like the cinema workshop at TsDKhVD framed maintaining ‘the closest and most frequent correspondence with the sphere of production’ as the cornerstone of their work and were tasked with delivering results that could assist the compilation of industry production plans.^133^ The effort made to engage industry professionals in the work of research centres is evident from attempts to disseminate research results beyond the narrow circle of pedologists and pedagogues at industry conferences and through publication in film journals and national newspapers.^134^ The existence of investigators who were at the same time filmmakers (including Nikolai Zhinkin) is another vivid indicator of meaningful interchange between research and production.

If the battle for a healthy cinema was liable to be frustrated by commercial considerations in NEP-era Russia – much like in the USA where activists failed to ensure the production of ‘edifying’ films despite the enforcement of more stringent censorship codes – the transformation of Soviet society and culture that began in the late 1920s brought Soviet film production into line with the recommendations of pedologists, psychologists and health professionals.^135^ The completion of the First Five-Year Plan and the surrender of profit making agendas to the imperative of cultivating a new type of citizen imbued the question of creating a suitable cinema for children with a new urgency. Indeed, the Soviet state's commitment to this costly undertaking came to be brandished as a badge of honour that affirmed Soviet culture's distinctiveness from its bourgeois counterpart.^136^ Alongside the establishment of the world's first film studio devoted to the creation of cinema for children (*Soiuzdetfil’m*, founded in 1936), however, the Stalin era saw the reorientation of the entire Soviet film industry towards a cinematic form that, to use the words of Sukharebskii, ‘instills a new joyfulness’, ‘extends the horizons of the psyche and enriches the personality’.^137^ Prizing accessibility, optimism, emotional appeal and pedagogical influence, the cinema that emerged after the advent of socialist realism effected the erosion of strict distinctions between productions for children and adults.^138^ The collaboration of pedologists, educators and industry professionals, while ostensibly targeting the creation of films specifically for children, contributed to the formation of a wider consensus on the characteristics of a ‘healthy’ cinema. By 1934, Soviet pedologists’ appeals for realism, clarity and continuity had become a requirement for all Soviet filmmakers.

## Footnotes

1 L.M. Sukharebskii, *Patokinographiia v psikhiatrii i nevropatologii* (Moscow 1936), 85, 193–4.

2 A. Piotrovskii, ‘Krest’iane: put’ k narodnoi tragedii’ [1934], in *Teatr. Kino. Zhizn’* (Leningrad 1969), 262–3; S. Dinamov, ‘Sozdadim iskusstvo strastnoe i mudroe’, *Rot-fil’m* (25 September 1934), 1–2.

3 Pivotal studies on the growing authority of medicine in Soviet Russia include: D. Beer, *Renovating Russia: The Human Sciences and the Fate of Liberal Modernity, 1880–1930* (Ithaca, NY 2008); F.L. Bernstein, *The Dictatorship of Sex: Lifestyle Advice for the Soviet Masses* (DeKalb, 2007); F.L. Bernstein, C. Burton and D. Healey, (eds), *Soviet Medicine: Culture, Practice, and Science* (DeKalb, IL, 2010), D. Healey, *Bolshevik Sexual Forensics: Diagnosing Disorder in the Clinic and the Courtroom, 1917–1939* (DeKalb, IL 2009); D. Healey, *Homosexual Desire in Revolutionary Russia: The Regulation of Sexual and Gender Dissent* (Chicago, IL 2001); I. Halfin, *Terror in My Soul: Communist Autobiographies on Trial* (Cambridge, MA 2003); D.L. Hoffmann: *Cultivating the Masses: Modern State Practices and Soviet Socialism, 1914–1939* (Ithaca, NY 2011); K.M. Pinnow, *Lost to the Collective: Suicide and the Promise of Soviet Socialism, 1921–1929* (Ithaca, NY 2010); S. Gross Solomon and J.F. Hutchinson (eds), *Health and Society in Revolutionary Russia* (Bloomington, IN 1990); T. Starks, *The Body Soviet: Propaganda, Hygiene, and the Revolutionary State* (Madison, WI 2008). On the cross-fertilization of science and culture during the 1920s, see N. Krementsov, *Revolutionary Experiments: The Quest for Immortality in Bolshevik Science and Fiction* (Oxford 2014); A. Banerjee, *We Modern People: Science Fiction and the Making of Russian Modernity* (Middletown, CT 2012).

4 For an example of pre-revolutionary calls for the need to transform the cinema into an instrument of enlightenment, see E. Samuilenko, *Kinematograph i ego prosvetitel'naia rol'* (St. Petersburg 1912).

5 International discussions concerning cinema's effects on children can be found in the journal *International Review of Educational Cinematography* (1929–34). Seminal secondary works on this topic include: A. Killen, *Homo Cinematicus: Science, Motion Pictures, and the Making of Modern Germany* (Philadelphia, PA 2017); K. Ritzheimer, *‘Trash’, Censorship, and National Identity in Early Twentieth-Century Germany* (Cambridge 2016); S. Curtis, *The Shape of Spectatorship: Art, Science, and Early Cinema in Germany* (New York, NY 2015); C. Ionita, ‘The Educated Spectator: Cinema and Pedagogy in France’, PhD dissertation, Columbia University (2013); A. Killen, *Berlin Electropolis: Shock, Nerves, and German Modernity* (Berkeley, CA 2006); A. Killen, ‘The Scene of the Crime: Psychiatric Discourses on the Film Audience in Early Twentieth-Century Germany’, *Harvard Review of Psychiatry*, 14, 1 (2006), 38–43; G. Jowett, I. Jarvie and K. Fuller, *Children and the Movies: Media Influence and the Payne Fund Controversy* (Cambridge 1996); S. Hake, *The Cinema's Third Machine: Writing on Film in Germany, 190–1933* (Lincoln, NE 1993).

6 ‘Introduction’, *International Review of Educational Cinematography*, 1 (1929), 7–11.

7 L. Dop, ‘The Role and the Purpose of the International Educational Cinematographic Institute’, *International Review of Educational Cinematography*, 1 (1929), 22.

8 K. Clark, *Petersburg: Crucible of Cultural Revolution* (Cambridge, MA 1995), 164.

9 N. Bukharin, *Kommunisticheskoe vospitanie molodezhi* (Moscow 1925), 71–2. L. Trotsky, *Problems of Everyday Life: Creating the Foundations for a New Society in Revolutionary Russia* (New York, NY 1973), 76.

10 L. Ritzheimer, ‘Protecting Youth from “Trash”: Anti-Schund Campaigns in Baden, 1900–1933’, PhD dissertation, State University of New York (2007), 74. On the ‘explosion of interest in childhood’ in Russia at the turn of the century, see C.Kelly, *Children's World: Growing Up in Russia, 1890–1991* (New Haven, CT 2007), 25–60.

11 E.M. Balashov, *Pedologiia v Rossii v pervoi treti XX veka* (St. Petersburg 2012). See also, A. Byford, ‘Parent Diaries and the Child Study Movement in Late Imperial and Early Soviet Russia’, *The Russian Review*, 72, 2 (April 2013), 212–41; A. Byford, ‘V. M. Bekhterev in Russian Child Science, 1900s–1920s: “Objective Psychology”/ “Reflexology” as a Scientific Movement’, *Journal of the History of the Behavioral Sciences*, 52, 2 (Spring 2016), 99–123.

12 See, for example: A. Gel’mont, *Chem zaniat den’ pionera i shkol’nika* (Moscow 1927) 34; A. Gel’mont ‘Voprosy organizatsii detskogo dosuga’, in A. Gel’mont and A. Durikin (eds), *Trud i dosug rebenka*, (Moscow 1927), 16; Gar et al., ‘Detskii dosug’, in *Trud i dosug rebenka*, 32–40.

13 V.A. Pravdoliubov, ‘Kino i uchashchiesia’, *Na putiakh k novoi shkole’*, 2 (1927), 22; V.A. Pravdoliubov, *Kino i nasha molodezh’: Na osnove dannykh pedologii* (Moscow-Leningrad 1930), 8–9. A.M. Gel’mont, ‘Kino i vospitanie’ in A. M. Gel’mont (ed.) *Kino-deti-shkola* (Moscow 1929), 8. Similar findings were obtained by a 1927 investigation into children's viewing habits conducted by the Central Cabinet of Pioneers. See Batashov, ‘Kino-utrenniki’, *Vozhatyi*, 1 (1927) 25–7.

14 Pravdoliubov, *Kino i nasha molodezh'*, 58.

15 Ibid., 59.

16 A. Zaikov, ‘Kinoopasnost’ i bor’ba s neiu’, *Na putiakh k novoi shkole*, 2 (1927), 13–18. Much in the same way, Brezhnev-era anxieties about ‘telemania’ and ‘television sickness’ also centred on children. See K. Roth-Ey, *Moscow Prime Time: How the Soviet Union Built the Media Empire that Lost the Cultural Cold War* (Ithaca, NY 2011), 206–8.

17 *Zdanie shkoly, pitanie, odezhda, rezhim truda i otdykha* (Moscow 1926), 60–1. See also N. Semashko et al., ‘Pioner dolzhen byt’ zdorovym!*’*, in E. P. Solov’eva (ed.), *Pioner dolzhen byt’ zdorovym!* (1926), 4–8. On NEP-era efforts to study the population's health, see Pinnow, *Lost to the Collective*, 15–20, 46–9.

18 *Zdanie shkoly*, 69–72; Semashko et al., ‘Pioner dolzhen byt’ zdorovym!’, 11, 16; E.P. Radin, *Pioner, beregi svoe zdorov’e* (Moscow 1925), 6–7; E.P. Radin, ‘Kak dolzhen podrostok provodit’ vremia otdykha’, in A. Ia. Kedrus (ed.) *Gigiena podrostka* (Moscow-Leningrad 1925), 72–7.

19 L.M. Sukharebskii, ‘Kino i zdorov’e detei’, in *Kino-deti-shkola*, 27. On *kinematoftalmiia*, see Pravdoliubov, *Kino i nasha molodezh’*, 137. See also S.E. Sovetov, *Uchebnoe kino i zdorov’e uchashchikhsia* (Moscow 1933), 9–12.

20 A. Angelucci et al., ‘The Cinema and Eye-Sight: Effects on Children's Sight’, *International Review of Educational Cinematography*, 5 (1930), 583–602.

21 A. Latsis and L. Keilina, *Deti i kino* (Moscow 1928), 12–14.

22 D.J. Youngblood, *Movies for the Masses: Popular Cinema and Soviet Society in the 1920s* (Cambridge 1992), 14.

23 Gel’mont, ‘Kino i vospitanie’, 5–24.

24 Sukharebskii, ‘Kino i zdorov’e detei’, 27.

25 V. Pravdoliubov, ‘O iunom kinozritele’, *Izvestiia* (21 August 1933), 4.

26 ‘Protokoly zasedaniia sektsii pedagogiki massovykh vozdeistvii’, Central State Archive of the City of Moscow (TsGAM), f. 2194, op. 1, d. 145, l. 77.

27 Pravdoliubov, *Kino i nasha molodezh'*, 128.

28 On nervous exhaustion as a characteristically Soviet disease, see Bernstein, *The Dictatorship of Sex*, 26 and Pinnow, *Lost to the Collective*, 218.

29 Pravdoliubov, *Kino i nasha molodezh'*, 77.

30 Pravdoliubov, ‘O iunom kinozritele’. For an example of concerns raised about the dangers inherent to the cinematic medium abroad, see M. Bernalei, ‘Use and Abuse of the Cinema’, *International Review of Educational Cinematography*, 1 (1934), 55–61.

31 See, for example, Latsis and Keilina, *Deti i kino*, 14.

32 Youngblood, *Movies for the Masses*, 19–20, 50.

33 B.M. Kheifets, ‘Detskii kino-teatr kak forma vneshkol’nogo vospitaniia’, in *Kino-deti-shkola*, 67; Latsis and Keilina, *Deti i kino*, 14; Pravdoliubov, *Kino i nasha molodezh’*, 74–5, 127–8; Kheifets, ‘Detskii kinoteatr’, 67–8.

34 Pravdoliubov, *Kino i nasha molodezh'*, 72.

35 Ibid., 73.

36 Pinnow, *Lost to the Collective*, 190, 228. On NEP-era laments about the corruption of Soviet young people and their typical framing of political threats in medical terms, see E. Naiman, *Sex in Public: The Incarnation of Early Soviet Ideology* (Princeton, NJ 1997), 262; Halfin, *Terror In My Soul*, 97–103. See also: Starks, *The Body Soviet*, 24–6; Bernstein, *The Dictatorship of Sex*, 37–8, 142–5. On the origins of Soviet anxieties about the health of children and youth in the late imperial period, see S.K. Morrissey, ‘The Economy of Nerves: Health, Commercial Culture, and the Self in Late Imperial Russia’, *Slavic Review*, 69, 3 (Fall 2010), 645–675.

37 Gel’mont, ‘Kino i vospitanie’, 5–6.

38 This trend is also noted by Latsis and Keilina. See their *Deti i kino*, 24.

39 Pravdoliubov, *Kino i nasha molodezh*', 64. ‘Sinking deep into the minds of our youth’, Pravdoliubov warned, ‘cinema shapes consciousness according to its own image, transforming the world-view and entire personality of the film viewer’ (65).

40 See the press citations compiled in Latsis and Keilina, *Deti i kino*, 16–17, and Iu. Menzhinskaia, ‘O kino dlia detei’, *Narodnoe prosveshchenie*, 3 (1928), 114–19.

41 M. Beliaev, ‘Deti i kino’, *Pravda* (6 January 1928), 4.

42 Latsis and Keilina, *Deti i kino*, 20; N.A. Tolstova, *Kino i deti* (Moscow 1930), 3.

43 N.F. Leisher, ‘O detskoi fil’me’, *Iskusstvo v shkole*, 5 (1928), 17; A. Zaikov, ‘Kinoopasnost’ i bor’ba s neiu’, *Na putiakh k novoi shkole*, 2 (1927), 13–18, 13; Latsis and Keilina, *Deti i kino*, 16–17.

44 *Vecherniaia Moskva*, 6 January 1927, cited in Latsis and Keilina, *Deti i kino*, 17.

45 Latsis and Keilina, *Deti i kino*, 16. As Scott Curtis notes, ‘For many physicians and reformers, cinema acted as a hypnotist, sending impressionable subjects to the streets with powerful, posthypnotic suggestions to commit crimes of all varieties’. See his *The Shape of Spectatorship*, 135.

46 Article in *Krasnyi put'*, 4 December 1927, quoted in A. Latsis and L. Keilina, *Deti i kino*, 17.

47 Kheifets, ‘Detskii kino-teatr’, 67.

48 A.E. Gorsuch, *Youth in Revolutionary Russia: Enthusiasts, Bohemians, Delinquents* (Bloomington IN 2000), 127.

49 P.I. Liublinskii, *Kinematograph i deti* (Moscow 1925), 18.

50 Quoted in V. Vainshtok and D. Iakobzon, *Kino i molodezh'* (Moscow-Leningrad 1926), 10.

51 Liublinskii, *Kinematograph i deti*, 20.

52 Iu. Menzhinskaia, ‘Blizhaishie zadachi v oblasti kino dlia detei’, *Na putiakh k novoi shkole*, 3 (1927), 6–7. See also, Liublinskii, *Kinematograph i deti*, 20.

53 Batashov, ‘Deti i kino’, *Vozhatyi*, 16 (1926), 38–40.

54 Latsis and Keilina, *Deti i kino*, 13. On the urban metropolis as space of capitalist temptation, see Starks, *The Body Soviet*, 16–17.

55 Tolstova, *Kino i deti*, 3.

56 N. Semashko, ‘Kino i zdravokhranenie’, *Kino i kul’tura*, 5–6 (1929), 25–6.

57 S. Curtis, *The Shape of Spectatorship*, 153–4.

58 Sukharebskii, ‘Kino i zdorov’e detei’, 30.

59 On the scientific and medical community's involvement in the transformation of cinema and its public in Germany, see Killen, *Homo Cinematicus*, 87.

60 For an overview of the Bolsheviks’ ideas on cultural revolution, see D.L. Hoffmann, *Stalinist Values: The Cultural Norms of Soviet Modernity, 1917–1941* (Ithaca, NY: 2003), 38-45. On the Soviet avant-garde's commitment to ‘the production of the new person by means of art’, see S. Tret’iakov, ‘Otkuda i kuda?: Perspektivy futurizma’, *Lef*, 1 (1923), 192–203.

61 A.M. Gel’mont, ‘Kino kak faktor vospitaniia’, *Vestnik prosveshcheniia*, 5 (1927), 9.

62 Liublinskii, *Kinematograf i deti*, 81; See also Gel’mont, ‘Kino kak faktor vospitaniia’, 10.

63 Tearing ‘film-maniacs’ away from the screen was also not an easy task; it was not uncommon for groups of teenagers to loudly protest their eviction from the cinema, leading to interrupted showings and police call outs. See Liublinskii, *Kinematograph i deti*, 82.

64 Ibid., 83.

65 *Rabochaia gazeta*, 28 May 1927, quoted in Iu. Menzhinskaia, ‘O kino dlia detei”, *Narodnoe prosveshchenie*, 3 (1928), 116.

66 D. Youngblood, ‘The Fate of Soviet Cinema during the Stalin Revolution’, *The Russian Review*, 50, 2 (1991), 157.

67 M. Krupenina, ‘Kino i deti’, *Na putiakh k novoi shkole*, 7–8 (1926), 38–40.

68 The Moscow Soviet's July 1928 decree, which ruled that children under 8 were only permitted to matinée screenings for children and that children under 16 could only attend general screenings before 9 o’clock in the evening was indicative of this approach. See ‘O poseshchenii det’mi kino-seansov v Moskve i Moskovskoi gubernii’, in *Kino-deti-shkola*, 66.

69 Iu. I. Menzhinskaia, ‘Massovaia kino-rabota s det’mi v Moskve i metody ee provedeniia’, in *Kino-deti-shkola*, 37–57; ‘Instruktsiia ob organizatsii pedagogicheskikh kino-utrennikov dlia detei v gor. Moskve’, *Ezhenedel’nik MONO*, 15–16 (1928), 4–6.

70 ‘Instruktivno-metodicheskie materially po voprosam vneshkol’noi raboty s det’mi’, Russian Academy of Education Archive (RAO), f. 5, op. 1, d. 193, l. 54.

71 G.A. Kister, *Detskii kinoteatr* (Moscow 1936), 31–2.

72 Batashov, ‘O kino-utrennikakh’, *Vozhatyi*, 19 (1926), 34.

73 Sukharebskii, ‘Kino i zdorov’e detei’, 34–5.

74 Kheifets, ‘Detskii kino-teatr’, 73.

75 L. Nikolaeva and N. Piatnitskaia, *Vneshkol’naia kinorabota s det’mi i podrostkami* (Moscow-Leningrad 1931), 10–16; Kheifets, ‘Detskii kino-teatr’, 73; Menzhinskaia, ‘Massovaia kino-rabota’, 49–52.

76 Kister, *Detskii kinoteatr*, 13.

77 As Kristin Roth-Ey's analysis of the anxieties that the medium of television sparked during the Brezhnev era has shown, passive absorption was anathema to the Soviet vision of cultured cultural consumption. See her *Moscow Prime Time*, 200–8.

78 Menzhinskaia, ‘Massovaia kino-rabota’, 37–57.

79 Kister, *Detskii kinoteatr*, 21–4.

80 ‘Sezon raboty pervogo detskogo kino-teatra’, in *Kino-deti-shkola*, 86–92; ‘Instruktsiia ob organizatsii’ 4–6; *Zadachi, sistema i metody vneshkol’noi kino-raboty s det’mi* (Leningrad 1933), 10–15; RAO, f. 5, op. 1, d. 193, l. 54ob; Kheifets, ‘Detskii kino-teatr’, 70–6.

81 ‘Sezon raboty’, 97. See also Kheifets, ‘Detskii kino-teatr’, 67–80.

82 N. Tolstova, ‘“Oktiabr’” u detei’, *Kino-deti-shkola*, 113–25.

83 ‘Stenogramma rasshirennogo vyezdnogo zasedaniia Vneshkol’nogo Soveta Mosgorono’, TsGAM, f. 528, op. 1, d. 76, l. 11. The network of children's film theatres in Soviet Russia had expanded to 46 by the mid–1930s. See Kister, *Detskii kinoteatr*, 3.

84 Pravdoliubov, ‘Kino i uchashchiesia’, 19.

85 A.A. Grigor’eva, ‘Kino v shkolakh Moskvy’, in *Uchebnoe i nauchnoe kino* (Leningrad 1947), 51; ‘Postanovlenie Sovnarkoma RSFSR o kinofikatsii massovoi shkoly’ (1932) in *Sbornik ofitsial’nykh postanovlenii i rasporiazhenii po voprosam khudozhestvennogo vospitaniia detei* (Moscow 1933), 8–9.

86 ‘O kabinete detskogo shkol’nogo uchebnogo kino’, RAO, f. 40, op. 2, d. 1030, ll. 50–56. By the end of the decade, nearly all schools in Moscow were equipped with film cameras and projectors. See Grigor’eva, ‘Kino v shkolakh Moskvy’, 51–2.

87 Gel'mont, ‘Kino kak faktor’, 9.

88 Latsis and Keilina, *Deti i kino*, 36, 41.

89 On the impact of the First Five-Year Plan on the Soviet film industry, see R. Taylor, ‘Ideology as Mass Entertainment: Boris Shumyatsky and Soviet Cinema in the 1930s’, in R. Taylor and I. Christie (eds), *Inside the Film Factory* (London 1991), 195–201; V. Kepley Jr., ‘The First “Perestroika”: Soviet Cinema under the First Five-Year Plan’, *Cinema Journal*, 35, 4 (Summer 1996), 31–53.

90 Pravdoliubov, ‘Kino i uchashchiesia’, 20; A. Gel’mont, ‘Kino i zadachi pedagogiki’, in *Detskoe kino* (Moscow 1930), 6.

91 On Soviet studies of film spectators during the 1920s, see: A. Toropova, ‘Probing the Heart and Mind of the Viewer: Scientific Studies of Film and Theatre Spectators in the Soviet Union, 1917–1936’, *Slavic Review*, 76, 4 (Winter 2017), 931–958; I.U. Fokht-Babushkin, ‘Izuchenie funktsionirovaniia kino vremen velikogo nemogo’, in I.U. Fokht-Babushkin (ed.), *Publika kino v Rossii: Sotsiologicheskie svidetel’stva 1910-1930-kh godov* (Moscow 2013), 1–50; M.N. Luk’ianova, ‘Osnovnye etapy sotsiologicheskoi razrabotki problemy kinoauditorii v Rossii’, in O.B. Bozhkov (ed.) *Sotsiologiia vchera, segodnia, zavtra*, (St. Petersburg 2012), 265–84; A. Olenina, ‘Psychomotor Aesthetics: Conceptions of Gesture and Affect in Russian and American Modernity, 1910s–1920s’, PhD dissertation, Harvard University, (2012); V. Bohlinger, ‘Engrossing! Exciting! Incomprehensible? Boring! Audience Survey Responses to Eisenstein's *October*’, *Studies in Russian and Soviet Cinema*, 5, 1 (2011), 5–27; A. Nesbet, *Savage Junctures: Sergei Eisenstein and the Shape of Thinking* (London 2007); N. Khrenov, ‘K probleme sotsiologii i psikhologii kino 20-x godov’, *Voprosy kinoiskusstva*, vyp. 17 (Moscow 1976), 163–84.

92 ‘Protokoly zasedanii kinokomiteta’, Central State Archive of the Literature and Art of St. Petersburg (TsGALI SPb), f. 82, op. 3, d. 20, ll. 103–4.

93 ‘Spravka, vydannaia Laboratoriei po izucheniiu mass’, Russian State Archive of Literature and Art (RGALI), f. 1923, op. 1, d. 2405, l. 1.

94 TsGALI SPb, f. 82, op. 3, d. 20, l. 97.

95 W.W. Charters, *Motion Pictures and Youth* (New York, NY 1933), vi; ‘Introduction’, *International Review of Educational Cinematography*, 1 (1929), 7.

96 Gel'mont, ‘Kino i zadachi pedagogiki’, 6–8.

97 ‘Protokoly zasedanii sektsii “pedagogika massovogo vozdeistviia” pri IMShR’, TsGAM, f. 2194, op. 1, d. 146, ll. 3-4; Pravdoliubov, *Kino i nasha molodezh*', 18–53; E. Stanchinskaia-Rozenberg, ‘Vliianie kino na shkol’nika’, *Vestnik prosveshcheniia*, 2 (1927), 8–25; A.M. Gel’mont, ‘Izuchenie vliania kino na detei (problema i metody)’, *Kul’tura i kino*, 4 (1929), 38–46.

98 ‘Materialy o rabote otdelov instituta, 1926–1928’, TsGAM, f. 2194, op. 1, d. 68, l. 33.

99 Gel’mont, ‘Izuchenie vliania kino’, 41.

100 Charters, *Motion Pictures and Youth*, 25.

101 ‘Otchet o rabote pedologicheskogo otdela za 1928/29gg’, TsGAM, f. 2194, op. 1, d. 116, l. 12; Gel’mont, ‘Izuchenie vliania kino’, 38–46.

102 Pravdoliubov, ‘O iunom kinozritele’; Gel’mont, ‘Izuchenie vliania kino na detei’, 45.

103 Gel’mont, ‘Izuchenie vliania kino na detei’, 44.

104 According to Nechaev, a lack of synchronicity between the results of the sensory and motor skills tasks signalled a child's exhaustion. See A.P. Nechaev, *Psikhologiia fizicheskoi kul'tury* (Moscow-Leningrad 1930), 73–80.

105 Pravdoliubov, *Kino i nasha molodezh'*, 127.

106 The 5-minute test required students to cross out specified numbers from a sheet of random figures. ‘Protokoly zasedaniia sektsii pedagogiki massovykh vozdeistvii’, TsGAM, f. 2194, op. 1, d. 145, l. 77.

107 ‘Otchetnye materialy instituta za 1927-1931’, RAO, f. 5, op. 1, d. 9, l. 128.

108 N.I. Zhinkin, “K voprosu o metodike postroeniia uchebnoi fil'my”, in *Detskoe kino*, 16.

109 ‘Materialy po organizatsionnym voprosam’, RAO, f. 5, op. 1, d. 3, l. 6; ‘Proizvodstvennyi plan kino-komissii na 1929-1930’, RAO, f. 5, op. 1, d. 9, ll. 306–7.

110 ‘M.A. Polman, “Doshkol’niki i kino, tom 1”’, RGALI, f. 2900, op. 1, d. 220, ll. 1, 7-7ob; ‘M. A. Polman, “Doshkol’niki i kino, tom 2”’, RGALI, f. 2900, op. 1, d. 221, ll. 1–10.

111 RGALI, f. 2900, op. 1, d. 220, ll. 3-5; RGALI, f. 2900, op. 1, d. 221, l.61.

112 The parents of those children who did not stay at the kindergarten overnight were asked to fill out a report on their son's or daughter's behaviour, activities, appetite and sleep patterns following the film showing. RGALI, f. 2900, op. 1, d. 220, l. 43; RGALI, f. 2900, op. 1, d. 221, l. 41, 43–5, 49. The Payne Fund researchers also led investigations into cinema's impact on sleep patterns. See Chambers, *Motion Pictures and Youth*, 31–5.

113 RGALI, f. 2900, op. 1, d. 220, l. 2. RGALI, f. 2900, op. 1, d. 221, ll. 1, 9–10.

114 For a summary of the Payne Fund's findings, see Chambers, *Motion Pictures and Youth*, 35.

115 RGALI, f. 2900, op. 1, d. 221, l. 50.

116 Despite the fact that the initiator of the Payne Fund project sought to produce irrefutable ‘scientific proof’ of cinema's negative impact on children and instigate tighter censorship laws, the researchers conducting the project were not activists for cinema reform. See Jowett et al., 8–9.

117 A. Shein, ‘Problema vospriiatiia i osmyshleniia kinofil’my det’mi’, *Tezisy dokladov k soveshchaniiu po voprosam massovogo vozdeistviia na VII mezhdunarodnoi psikhotekhnicheskoi konferentsii* (Moscow-Leningrad 1931), 22–4. So staunch was Soviet researchers’ commitment to the task of unleashing cinema's prophylactic power that those individuals whose findings cast doubt on the viability of this enterprise risked denunciation as ‘cinema pessimists’. The leader of the IMShR's research project on fatigue and film watching, Pravdoliubov, was ultimately replaced by a new investigator who pursued a different methodology and obtained results that were more favourable to cinema. To be sure, Pravdoliubov's findings were not withheld from publication. They were, however, fiercely criticized by other pedologists who argued that his line of work was harmful to the cause of establishing a healthy cinema for children. See, ‘Stenogramma zasedaniia sektsii uchebnogo kino NIS po obsuzhdeniiu raboty V. A. Pravdoliubova’, RGALI, f. 2900, op. 1, d. 118, ll. 1–7.

118 The 1936 decree against ‘pedological distortions’ decried ‘pseudo-scientific experiments’, ‘senseless and harmful questionnaires’ and ‘tests’. See R. Bauer, *The New Man in Soviet Psychology* (Cambridge, MA 1952), 123–4.

119 ‘Nauchno-issledovatel'skaia rabota N. Zhinkina i S. P. Vinogradova “Issledovanie effektivnosti shkol’no-uchebnogo fil’ma”’, RGALI, f. 2900, op. 1, d. 193. Sections of Zhinkin's work at VGIK were published: ‘Izuchenie zritelia i problemy postroeniia uchebnoi fil’my’, *Uchebnoe kino*, 6 (1934), 14–25; ‘Elementy siuzhetnosti v uchebnom fil’me’, *Uchebnoe kino*, 1 (1936), 7–20; ‘Neudachnye mesta shkol’no-uchebnykh fil’mov’, *Uchebnoe kino*, 3 (1936), 26–36.

120 RGALI, f. 2900, op. 1, d. 118.

121 ‘O kabinete detskogo shkol’nogo uchebnogo kino’, RAO, f. 40, op. 2, d. 1030, l. 2.

122 RAO, f. 40, op. 2, d. 1030, ll. 2–4, 42–4, 45–8, 69–72; ‘Plan raboty TsDKhVD na 1932’, RAO, f. 40, op. 1, d. 5, ll. 2-2ob; ‘Protokoly zasedaniia direktsii TsDKhVD’, RAO, f. 40, op. 1, d. 1, ll. 89–94. ‘Establishing hygienic film demonstration’ and combating ‘the anti-pedagogical influence of the existing cinema repertoire’ were also part of its duties. See RAO, f. 40, op. 2, d. 1030, l. 2.

123 RAO, f. 40, op. 2, d. 1030, ll. 73–5.

124 RGALI, f. 2900, op. 1, d. 221, ll. 6–7; N. Arnol’d, T. Kiselev and M. Polonskii, ‘Ispol’zovanie kino v nachal’noi shkole’, in B. S. Peres (ed.) *Shkol’no-uchebnyi fil’m* (Moscow 1935), 48–9; I. Menzhinskaia, ‘Kogo vospityvaet sovetskaia kinematografiia’, in *Detskoe kino*, 32.

125 Zhinkin, ‘K voprosu o metodike postroeniia uchebnoi fil’my’, 16. See also T. Kiselev, ‘Kak shkol’nik vosprinimaet fil’m’, *Uchebnoe kino*, 5 (1936), 23–4, 28.

126 Tolstova, ‘“Oktiabr’” u detei’, 122–123.

127 RGALI, f. 2900, op. 1, d. 221, l. 7. See also Arnol’d, Kiselev and Polonskii, ‘Ispol’zovanie kino’, 47–8.

128 ‘The action should develop logically’, Pravdoliubov noted, ‘without any startling jumps, without shifts from one place to the other and any muddling of the chief dramatic line by parallel plots. The entire picture must be unified and developed to the end’. See his *Kino i nasha molodezh’*, 92. See also: N.I. Zhinkin, ‘Izuchenie detskogo otnosheniia k kinematograficheskoi kartine’, *Pedologiia*, 4 (1930), 505–18; Zhinkin, ‘K voprosu o metodike’, 16; Zhinkin, ‘Elementy siuzhetnosti’; Zhinkin, ‘Neudachnye mesta’. As Evgeny Steiner has shown, 1920s reader studies similarly uncovered a dislike of fragmentation and abstraction among young Soviet readers. See his *Stories for Little Comrades: Revolutionary Artists and the Making of Early Soviet Children's Books* (Seattle, WA and London 1999), 46.

129 Zhinkin, ‘K voprosu o metodike’, 11.

130 Pravdoliubov, *Kino i nasha molodezh'*, 92–3.

131 Ibid., 129.

132 ‘Proizvodstvennyi plan kino-kabineta na 31/32 god’, RAO, f. 40, op. 3, d. 1030, l. 89.

133 ‘Tipovoe polozhenie’, RAO, f. 40, op. 3, d. 1030, ll. 1; RAO, f. 40, op. 3, d. 1030, l. 89. See also ‘Sviaz’ IMVR s drugimi uchrezhdeniiami i organizatsiiami’, RAO, f. 5. Op. 1, d. 3, ll. 1–10.

134 ‘Proizvodstvennyi plan kino-kabineta na 31/32 god’, l. 93. Institutes like the IMVR even sought to circulate their findings abroad, making contact with the American Society of Cinematographers. See RAO, f. 5, op. 1, d. 2, l. 90.

135 Reluctant to attribute the enforcement of the Motion Picture Production Code in 1934 on the dissemination of the Payne Fund studies, Jowett, Jarvie and Fuller argue that the ‘cleanup’ of the US film industry after 1934 was ‘more superficial than real’. See their *Children and the Movies*, 109.

136 N. Riabchikova, ‘Children's Cinema in the 1920s’, *Studies in Russian and Soviet Cinema*, 3, 2 (2009), 231–5. ‘Soviet cinema is the first in the history of world cinema to implement the organisation of the production of films for children’, boasted the industry head, Boris Shumiatskii in 1934. ‘The cinema of capitalist countries’, he continued, ‘completely dismisses the production of films for children as unprofitable’. See ‘Report from B.Z. Shumiatskii to CC RCP (b) on the Work of GUKF in the Area of Children's Cinema’, 14 September 1935, *Studies in Russian and Soviet Cinema*, 3, 2 (2009), 250–3, 251.

137 Sukharebskii, *Patokinographiia v psikhiatrii i nevropatologii*, 194. On the development of *Soiuzdetfil’m*, see Jeremy Hicks, ‘Soiuzdetfilm: The Birth of Soviet Children's Film and the Child Actor’, in B. Beumers (ed.), *A Companion to Russian Cinema* (Chichester 2016), 141–61.

138 Many films produced by *Soiuzdetfil’m*, including *Iakov Sverdlov* (Iutkevich 1940), *Zoia* (Arnshtam 1944) and *The Village School Teacher* (*Sel’skaia uchitel’nitsa*, Donskoi, 1947) came to be regarded as classics of socialist realism rather than films for children. For a similar argument in reference to socialist realist literature, see L. Heller, ‘A World of Prettiness: Socialist Realism and its Aesthetic Categories’, in T. Lahusen and E. Dobrenko (eds), *Socialist Realism Without Shores* (Durham, N.C 1997), 65–6.

